# Letter to the Editor from Hirschler: “Mauriac syndrome: automated insulin delivery treatment or adherence?”

**DOI:** 10.1210/jcemcr/luag057

**Published:** 2026-05-22

**Authors:** Valeria Hirschler

**Affiliations:** Department of Education and Research, Coordinator of the Research Committee (CIDEI), Sociedad Argentina de Diabetes, Buenos Aires C1006, Argentina

Regarding the article “Mauriac Syndrome: Growth and Clinical Outcomes After 2.5 Years of Automated Insulin Delivery Treatment” [1], I would like to emphasize the case's clinical relevance and share observations from our experience in a resource-limited setting.

Classic Mauriac syndrome is now uncommon; however, significant growth delay related to chronic insulin deficiency is still frequently observed in Latin American centers [2]. Importantly, our patient population differs substantially from that described by the authors. We predominantly work in public healthcare settings in Latin America, where families often face major barriers to diabetes care, including limited access to glucose test strips and continuous glucose monitoring. Moreover, automated insulin delivery (AID) systems are extremely limited and, in most cases, practically unattainable in routine clinical practice.

An especially notable detail in the case described by the authors is the patient's high level of adherence and engagement with medical follow-up prior to initiating AID. The patient attended clinic visits and completed multiple diagnostic evaluations, including a liver biopsy. This contrasts with what we typically observe in Mauriac-like cases in Latin American settings, where patients frequently miss appointments and show poor adherence to insulin therapy. It is also striking that the patient adhered to growth hormone therapy. This highlights the critical influence of behavioral and family factors on clinical outcomes.

This case prompts a central clinical question: Is the observed improvement in outcomes primarily attributable to the implementation of AID technology or to the preexisting acceptable level of family engagement and adherence?

Similarly, was it the device itself that “rescued” the Mauriac phenotype, or was it the enhanced professional attention and support provided to the family?

Most likely, the outcome reflects the synergistic interaction of all these elements: technology, adherence, family involvement, and professional guidance.

This case, therefore, illustrates not only the value of AID but also the central importance of continuity of care and family engagement, which in low-resource settings often determine outcomes even more strongly than access to advanced technology.

## Disclosures

The author reports no conflicts of interest.

## Data Availability

No new data were generated or analyzed in support of this research.
